# Time spent at blood pressure target and the risk of death and cardiovascular diseases

**DOI:** 10.1371/journal.pone.0202359

**Published:** 2018-09-05

**Authors:** Sheng-Chia Chung, Mar Pujades-Rodriguez, Bram Duyx, Spiros C. Denaxas, Laura Pasea, Aroon Hingorani, Adam Timmis, Bryan Williams, Harry Hemingway

**Affiliations:** 1 Health Data Research UK London, University College London, London, United Kingdom; 2 Institute of Health Informatics, University College London, London, United Kingdom; 3 Leeds Institute of Biomedical and Clinical Sciences, University of Leeds, Leeds, United Kingdom; 4 CAPHRI School for Public Health and Primary Care, Maastricht University, Maastricht, the Netherlands; 5 Institute of Cardiovascular Science, University College London, London, United Kingdom; 6 National Institute for Health Research (NIHR), University College London Hospitals Biomedical Research Centre, London, United Kingdom; 7 Barts Heart Centre, London, United Kingdom; International University of Health and Welfare, School of Medicine, JAPAN

## Abstract

**Background:**

The time a patient spends with blood pressure at target level is an intuitive measure of successful BP management, but population studies on its effectiveness are as yet unavailable.

**Method:**

We identified a population-based cohort of 169,082 individuals with newly identified high blood pressure who were free of cardiovascular disease from January 1997 to March 2010. We used 1.64 million clinical blood pressure readings to calculate the TIme at TaRgEt (TITRE) based on current target blood pressure levels.

**Result:**

The median (Inter-quartile range) TITRE among all patients was 2.8 (0.3, 5.6) months per year, only 1077 (0.6%) patients had a TITRE ≥11 months. Compared to people with a 0% TITRE, patients with a TITRE of 3–5.9 months, and 6–8.9 months had 75% and 78% lower odds of the composite of cardiovascular death, myocardial infarction and stroke (adjusted odds ratios, 0.25 (95% confidence interval: 0.21, 0.31) and 0.22 (0.17, 0.27), respectively). These associations were consistent for heart failure and any cardiovascular disease and death (comparing a 3–5.9 month to 0% TITRE, 63% and 60% lower in odds, respectively), among people who did or did not have blood pressure ‘controlled’ on a single occasion during the first year of follow-up, and across groups defined by number of follow-up BP measure categories.

**Conclusion:**

Based on the current frequency of measurement of blood pressure this study suggests that few newly hypertensive patients sustained a complete, year-round on target blood pressure over time. The inverse associations between a higher TITRE and lower risk of incident cardiovascular diseases were independent of widely-used blood pressure ‘control’ indicators. Randomized trials are required to evaluate interventions to increase a person’s time spent at blood pressure target.

## Introduction

Long-term lifestyle and pharmacological interventions to lower blood pressure (BP) have been shown in randomized trials to be effective in reducing cardiovascular morbidity and mortality[1–5]but little is known about the time an individual patient from the general population spends with their BP controlled. Sub-optimal BP control to current management targets is common (19–61%) across different health systems.[7–10] With new guidelines recommend treating to lower target BP levels,[11] clinicians may face even greater challenges in achieving and sustaining long-term control, and managing treatment contraindications. Despite hypertension being a chronic condition, current clinical convention is to assess BP ‘control’ at a one-off time point, and guidelines do not make recommendations of the frequency of measurement, how repeated measures might be used, nor evaluating longitudinal control.[2,4,6] In trials, it is common to measure BP at 3–6 monthly intervals and achieve high levels of treatment compliance and control,[12–14] but the frequency of BP measures varies in usual care has not been well studied and is likely to be lower.

The ‘area under the time curve’ of BP level is associated with cardiovascular disease endpoints.[15] The concept of time at target using repeated measurements has application in other clinical areas such as warfarin monitoring with international normalized ratio.[16] Time at BP target might be an important measure of hypertension management effectiveness but one which has yet to be evaluated in the general population. Three questions remain unanswered: firstly, what is the average time in a year that BP remains at the management target in hypertensive patients in usual care settings? Secondly, how do patient characteristics and use of BP-lowering medications influence the time at BP target? Thirdly, to what extent is time at BP target, independent of other BP control indicators, such as a single measure of ‘control’ or visit-to-visit BP variability,[17,18] associated with clinical outcomes?[19,20]

We studied an English population because of national remuneration scheme to achieve the same BP lowering goal across general practices.[21] and because of availability of nationwide population-based linked electronic health records in which BP and disease endpoint data have been extensively validated.[1,22–25] We studied individuals with newly identified hypertension, which provides clinical opportunities for the early optimization of BP management to prevent cardiovascular diseases.

## Methods

The authors declare that all methods are available within the manuscript (and its online-only Supporting Information).

### Data sources

We used primary care records of individual patients from the 225 primary care practices contributing data to the Clinical research using LInked Bespoke studies and Electronic health Records (CALIBER) program during the study period from January 1997 to March 2010. CALIBER links patient records from four different data sources: primary care (Clinical Practice Research Datalink), disease registry (Myocardial Ischaemia National Audit Project registry), hospital care (Hospital Episodes Statistics), and death registry (Office of National Statistics). A description of CALIBER[22] and the phenotyping process[25] and extensive validations of blood pressure, other risk factors and cardiovascular endpoints have been previously published[1,23,24,26,27] (www.caliberresearch.org/portal). The study was approved by the Independent Scientific Advisory Committee of the Medicines and Healthcare products Regulatory Agency in accordance with the Declaration of Helsinki.

### Study population

Newly identified high BP was defined based on recorded values in primary care according to the most recent guidelines: ≥140 mmHg systolic blood pressure (or ≥150 mmHg for people aged ≥60 years without diabetes and chronic kidney disease) and/or ≥90 mmHg diastolic blood pressure. The majority of general practitioners use digital sphygmomanometers,[28] which have been shown to have an accuracy comparable to mercury devices. Individuals included had three or more systolic or diastolic BP measures meeting these criteria within a one-year period, and entered the cohort on the date of the last BP measurement. People were excluded; (i) if they had prior history of hypertension before study inclusion (hypertension diagnosis or prescription of BP-lowering medication), or (ii) if they had prior history of any of the cardiovascular diseases used as study endpoints, or (iii) follow-up of less than six months since the third BP-measure. Individuals eligible for study inclusion were eighteen years or older, and registered with their practices for at least one year.

### Main exposure: Time at target

The main exposure was the annual percent time BP at management target, averaged over follow-up years (in short: TIme at TaRgEt (TITRE)). The treatment goal was set to <140/90 mmHg or <150/90 mmHg for patients aged ≥60 years without diabetes and chronic kidney disease according to treatment guidelines.[29–31] TITRE calculation was based on BP values recorded after the diagnosis (study entry). Details of the TITRE calculation and algorithms are presented in S1 Method. In brief, each recorded BP value prior to the censoring date, was first defined as on target or not. When multiple BP measures were recorded on the same date, the mean value was used. Second, for every two consecutive recorded BP values, the duration in days when BP was at target was calculated. If the BP control status changed, the duration was calculated assuming a linear decrease (or increase) of BP over time.[32] For each individual, the TITRE was calculated by averaging annual percent time at target over all follow-up years (S1 Method). The first, second and third quartiles of TITRE were 2.7% (0.3 months), 23.5% (2.8 months), and 46.9% (5.6 months). As the difference between quartiles was about 3 months, we chose a 3-month interval for TITRE categories, and classified TITRE into six groups: missing, 0 time, less than 3 months, 3 to 5.9 months, 6 to 8.9 months, and 9 to 11.9 months.

### Other indicators of blood pressure management

At baseline we evaluated systolic and diastolic BP and stage-two hypertension status (systolic BP ≥160 mmHg or diastolic blood pressure ≥100 mmHg). On follow-up we evaluated lifestyle intervention: receiving diet advice or regimen (weight-loss, low-sodium or low-fat), and smoking cessation among people who reported at baseline as current or past smoking. Pharmaceutical interventions included first BP-lowering drug, the delay from study entry to prescription of first BP-lowering drug, snapshot control status defined as having a single on-target blood pressure measure within the first year of follow-up, and a binary follow-up control status defined as the mean follow-up BP below or exceeding the treatment target. We evaluated in sensitivity analyses other alternative BP control measures, such as the median absolute deviation for average BP (7.5 mmHg for SBP and 5 mmHg for DBP), BP visit-to-visit variability, and number of follow-up BP measures.

### Other covariates

Factors affecting cardiovascular risk were recorded during consultations in primary care practices. Baseline demographic factors included age, sex, year of study entry, ethnicity, and index of multiple deprivation quintiles.[33] Other baseline cardiovascular risk factors included smoking status, body mass index, diabetes mellitus, total cholesterol, and renal dysfunction (defined as a history of renal failure recorded in primary and hospital care, or an estimated glomerular filtration rate <60 mL/min/1.73 m^2^, based on the Modification of Diet in Renal Disease formula[34]). Other treatment covariates included statin and aspirin use at baseline.

### Endpoints

Individuals were followed up for the occurrence of incident non-fatal cardiovascular diseases and death in the linked records. The primary endpoints were a composite of cardiovascular death, non-fatal myocardial infarction and stroke (including ischemic stroke, intracerebral hemorrhage and sub-arachnoid hemorrhage); incident fatal and non-fatal heart failure; any cardiovascular disease and death (as above plus angina, atrial fibrillation, transient ischemic attack, peripheral artery disease, abdominal aortic aneurysm, other coronary heart disease and deaths). Secondary endpoints were incident stable angina, peripheral artery disease, and all-cause mortality. Non-cardiovascular mortality was included in the study as an outcome reflecting the general improvement of clinical care over time (negative control), and its association with TITRE was evaluated. Patients were censored on the date of the first occurrence of incident cardiovascular disease, death, de-registration or final data collection from practices.

### Statistical analysis

We summarized individual demographics, risk factors and BP management by categorical TITRE group using descriptive statistics. Differences between groups were tested with Chi-square, one-way ANOVA, or nonparametric Wilcoxon rank sums test, as appropriate.

A mixed model was used to investigate the association between study covariates and change in TITRE. To investigate the risk of cardiovascular disease with the TITRE, we used the categorical TITRE in generalized mixed effects models weighted for duration of follow-up. A 0% TITRE was chosen as the reference group. Outcome-specific models were weighted by follow-up years, and adjusted for other blood pressure management indicators, and other covariates previously described. The random effect of primary care practice was incorporated in each model. Multicollinearity of covariates was examined by collinearity diagnostics, and model fit evaluated by the area under the ROC curve obtained from the models. The Akaike’s information criteria was used to evaluate the prognostic power of TITRE. We evaluated the number of estimated deaths and cardiovascular events delayed or deferred, under the hypothetic scenario of patients experienced the case mix and treatment standardized event risk of one level higher TITRE category then their own (S3 Method).

To examine the effect of TITRE comparing with other indicators of blood pressure management, in the main analyses, we include the indicator of snapshot control status during the first year of follow-up. It was also the a priori subgroup covariate, and main analyses were performed among patients who did or did not achieve blood pressure control during the first year of follow-up. Sensitivity analyses included: 1) replacing TITRE with a) snapshot control status, b) binary control status derived from mean follow-up BP values, c) the averaged value, or d) standard deviation of follow-up systolic blood pressure measures; and 2) including in TITRE models additional adjustment for standard deviation of follow-up blood pressure measurements, or the number of follow-up blood pressure measures. Missing covariate values were limited and managed by multiple imputation (S4 Method). Analyses were performed with SAS (version 9.4), STATA (version 13.1), and R (version 2.9.2).

## Results

**Fig 1:**
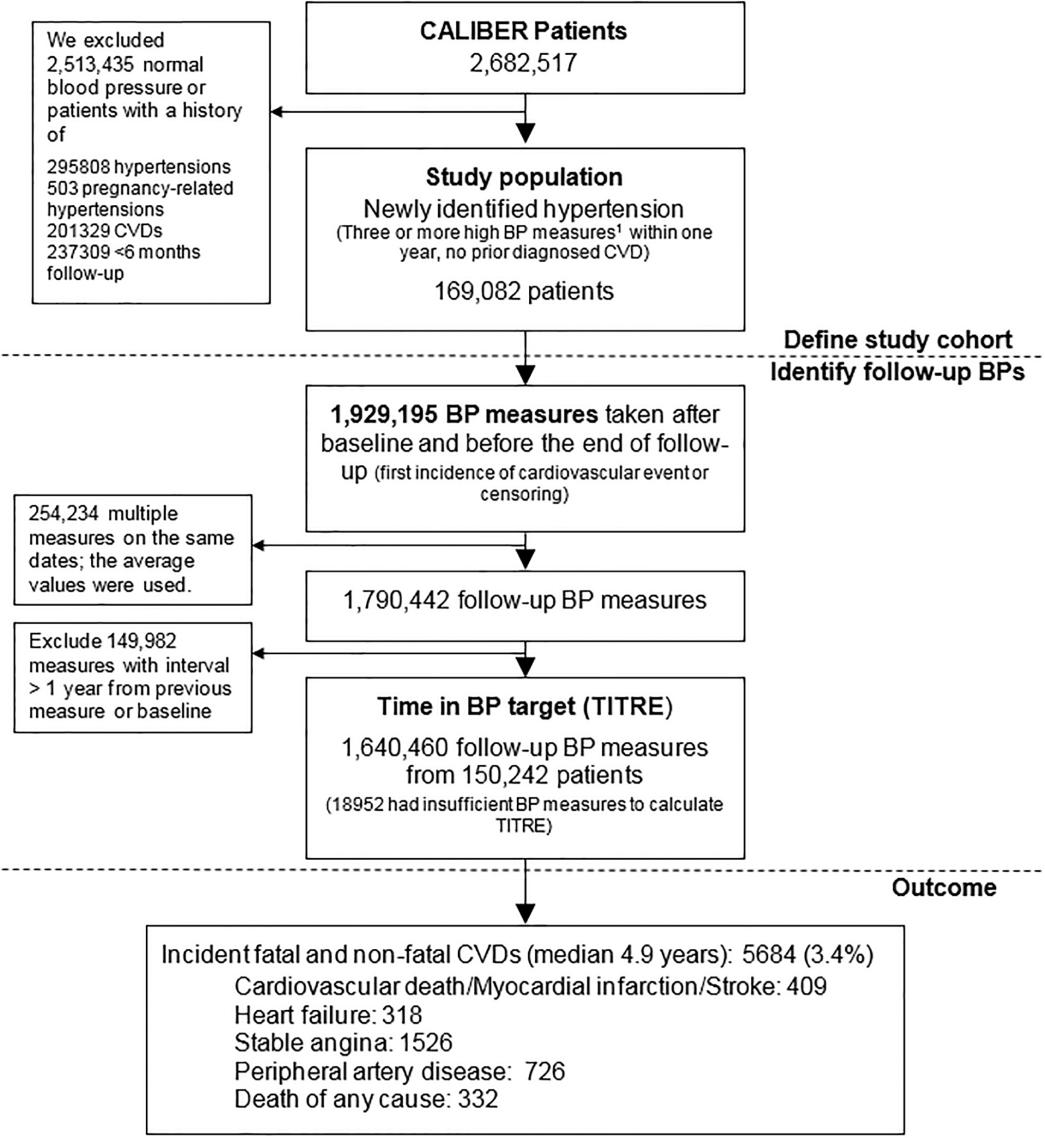
Definition of newly identified hypertension, average annual time at target (TITRE) and fatal and non-fatal cardiovascular events. ^1^ BP <140/90 mmHg, or <150/90 for patients aged 60 years or more without diabetes and chronic kidney disease. BP: blood pressure. CVD: cardiovascular disease. ^2^ For each patient, the days that blood pressure was at target between two consecutive measures were summed to calculate the percent time at target for each follow-up year, and then averaged over follow-up years. Details are described in S1 Method. ^3^ Incident cardiovascular disease include: myocardial infarction, stroke (including ischemic stroke, intracerebral hemorrhage and sub-arachnoid hemorrhage), heart failure, angina, atrial fibrillation, transient ischemic attack, peripheral artery disease, abdominal aortic aneurysm and cardiovascular and non-cardiovascular mortality.) It is unclear to what the (1) refers to in the figure.

We investigated 169,082 Individuals with newly identified hypertension (Fig 1), with mean age 51.5 years, and 55.7% women (Table 1). The median follow-up was 4.9 years (interquartile range: 2.6, 7.3 years). The prevalence of diabetes and stage-two hypertension were 4.8% and 38.4% at baseline. In the 45.7% patients who had prescriptions of one or more blood pressure lowering drugs during follow-up, thiazide diuretics or angiotensin-converting-enzyme inhibitor were the most frequent choice of initial drug treatment (16.0% and 15.4%, respectively). A record on dietary advice during follow-up was found in 28.6% of patients and smoking cessation in 2.0% of patients reporting current or former smoking at study entry.

**Table 1:** Patient characteristics in groups defined by time at target (TITRE)*.

Number of patients	All	Time at target (TITRE) categories					
		Unrecorded	0%	<3 months	3–5.9 months	6–8.9 months	9–11.9 months
	169082	18952	25866	51819	39651	25237	7557
Follow-up years	4.9 (2.6, 7.3)	2.8 (1.4, 5)	3.3 (1.6, 5.9)	5.1 (2.8, 7.5)	5.9 (3.5, 8.1)	5.9 (3.6, 8)	5.2 (3, 7.2)
**Blood pressure measurements and treatment**							
Average number of BP measures per year	1.6 (0.7, 2.8)	N/A	0.7 (0.3, 1.5)	1.4 (0.7, 2.5)	2 (1.1, 3)	2.3 (1.3, 3.2)	2 (1.3, 2.9)
Systolic BP at entry	150 (140, 160)	144 (140, 150)	150 (142, 160)	150 (142, 160)	150 (140, 161)	150 (140, 162)	148 (140, 160)
Diastolic BP at entry	90 (85, 96)	90 (82, 92)	91 (87, 98)	90 (85, 98)	90 (85, 97)	90 (85, 96)	90 (83, 95)
Stage two hypertension at entry	64974 (38.4)	3156 (16.7)	11105 (42.9)	21581 (41.6)	16467 (41.5)	10218 (40.5)	2447 (32.4)
Initial BP lowering drug use since study entry							
None	91748 (54.3)	17751 (93.7)	17990 (69.6)	25967 (50.1)	15680 (39.5)	10286 (40.8)	4074 (53.9)
Calcium channel blocker	12785 (7.6)	134 (0.7)	1467 (5.7)	4665 (9)	3889 (9.8)	2126 (8.4)	504 (6.7)
Thiazides diuretics	27014 (16)	197 (1)	1964 (7.6)	7843 (15.1)	9034 (22.8)	6534 (25.9)	1442 (19.1)
ACEI	26006 (15.4)	217 (1.1)	3266 (12.6)	9531 (18.4)	7649 (19.3)	4362 (17.3)	981 (13)
ARB	2234 (1.3)	22 (0.1)	223 (0.9)	725 (1.4)	728 (1.8)	418 (1.7)	118 (1.6)
Beta blocker	6704 (4)	425 (2.2)	713 (2.8)	2171 (4.2)	1902 (4.8)	1155 (4.6)	338 (4.5)
Other BP lowering drugs	3725 (2.2)	230 (1.2)	350 (1.4)	1241 (2.4)	1133 (2.9)	600 (2.4)	171 (2.3)
Delay from diagnosis to first drug use, years	0.7 (0.1, 2.9)	0.7 (0, 2.7)	0.4 (0, 2.4)	1.3 (0.1, 3.8)	0.8 (0.1, 3)	0.3 (0.1, 1.8)	0.1 (0, 0.6)
Number of BP lowering drug use^1^ during follow-up	0.0 (0.0,2.0)	0.0 (0.0,0.0)	0.0 (0.0,1.0)	0.0 (0.0,2.0)	1.0 (0.0,2.0)	1.0 (0.0,2.0)	0.0 (0.0,1.0)
Dietary advice during follow-up	48360 (28.6)	1970 (10.4)	5477 (21.2)	15756 (30.4)	13703 (34.6)	8992 (35.6)	2462 (32.6)
Snapshot ‘controlled’ within first year	79713 (47.1)	0 (0)	0 (0)	27936 (53.9)	24924 (62.9)	19831 (78.6)	7022 (92.9)
**Cardiovascular risk factors at study entry**							
Age (years), mean, SD	51.5, 14.0	48.2, 13.2	49.6, 12.3	51.8, 13.2	53.1, 14.3	53.3, 15.6	50.9, 16.9
Women	94242 (55.7)	8225 (43.4)	11469 (44.3)	28058 (54.1)	24687 (62.3)	16529 (65.5)	5274 (69.8)
White ethnicity	76846 (45.4)	7258 (38.3)	10027 (38.8)	23570 (45.5)	19461 (49.1)	12748 (50.5)	3782 (50)
Most deprived quintile	33733 (20)	3882 (20.6)	5361 (20.8)	10204 (19.8)	7803 (19.8)	4976 (19.8)	1507 (20)
Ex-smoker	33785 (23.7)	3891 (23.6)	5137 (23.3)	10446 (24.1)	7887 (24)	4873 (23.1)	1551 (24)
Current smoker	23021 (16.2)	3495 (21.2)	4251 (19.3)	6778 (15.6)	4671 (14.2)	2873 (13.6)	953 (14.8)
Body Mass Index	27.3(24.3, 31)	27.5(24.4, 31.2)	28(24.9, 31.8)	27.4(24.5, 31.2)	27(24.1, 30.8)	26.8(23.9, 30.5)	26.6(23.6, 30.1)
Diabetes	8185 (4.8)	208 (1.1)	835 (3.2)	2362 (4.6)	2507 (6.3)	1741 (6.9)	532 (7)
Renal dysfunction	7256 (4.3)	516 (2.7)	748 (2.9)	2086 (4)	2002 (5)	1465 (5.8)	439 (5.8)
Aspirin	2033 (1.2)	126 (0.7)	190 (0.7)	505 (1)	595 (1.5)	445 (1.8)	172 (2.3)
Statin	4987 (2.9)	342 (1.8)	569 (2.2)	1348 (2.6)	1377 (3.5)	1005 (4)	346 (4.6)

*Baseline values unless specified otherwise. Values are number and (%) for categorical variables; median and (first quartile, third quartile) for continuous variable, unless specified differently. P values for distributions of variables among all TITRE categories are all <0.001, besides for the ex-smoking (p = 0.0387) and most deprived quintile (p = 0.0035)

^1^included ACEI, angiotensive II antagonist, alpha blocker, centrally acting anti-hypertensive drugs. SD: standard deviation; BP: blood pressure; BP: blood pressure; renal dysfunction: history of renal failure or eGFR < 60 mL/min/1.73 m^2^; stage two hypertension: systolic pressure ≥160 mmHg or a diastolic pressure ≥100 mmHg. Dietary advice includes advice for low-sodium, weight-loss, or low-fat diet given during primary care consultation.

### TIme at target (TITRE)

**Fig 2:**
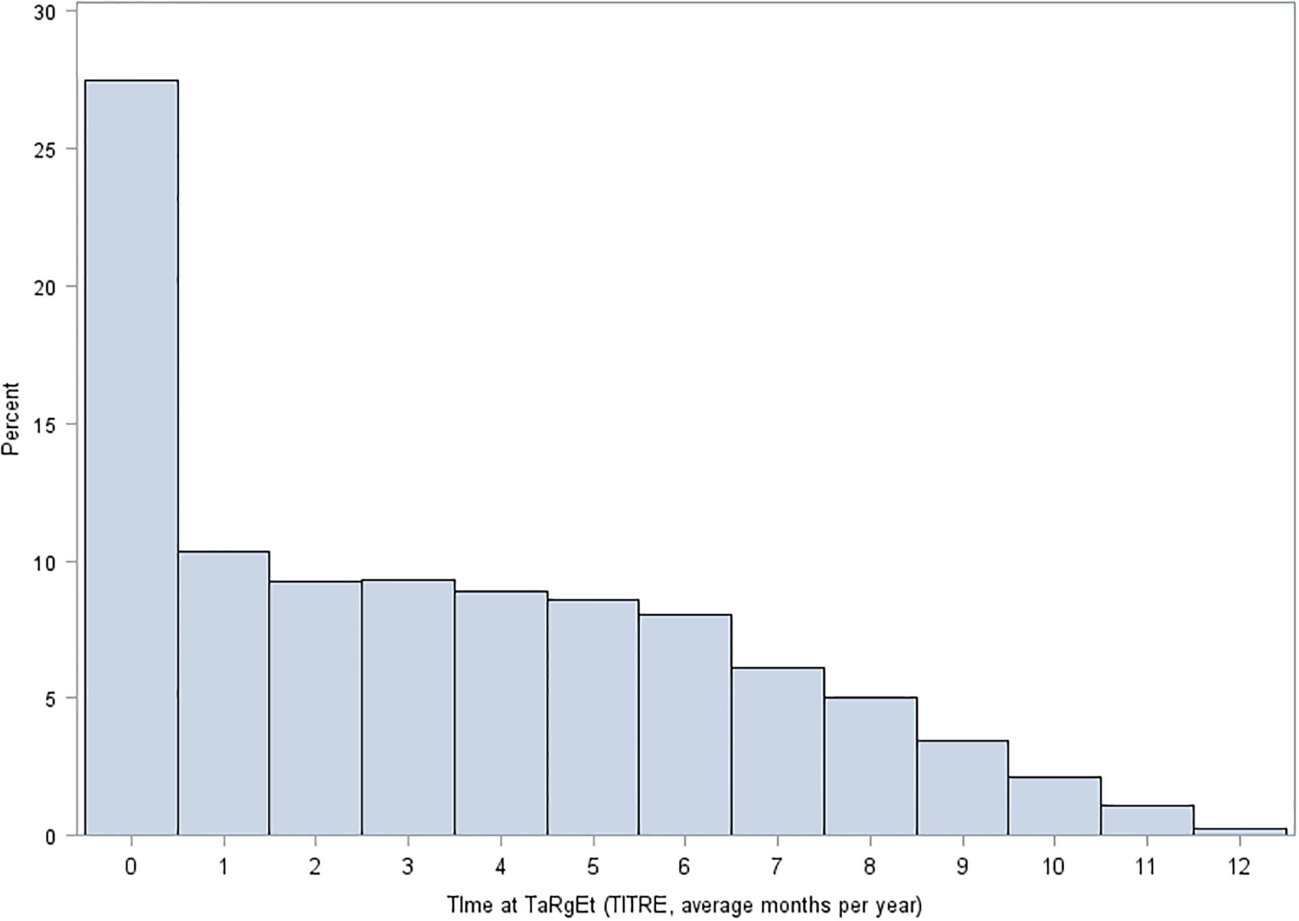
Time at target (TITRE) distribution in patients with recorded TITRE (N = 150130). *Median 2.8 (0.3, 5.6) months.

**Fig 3:**
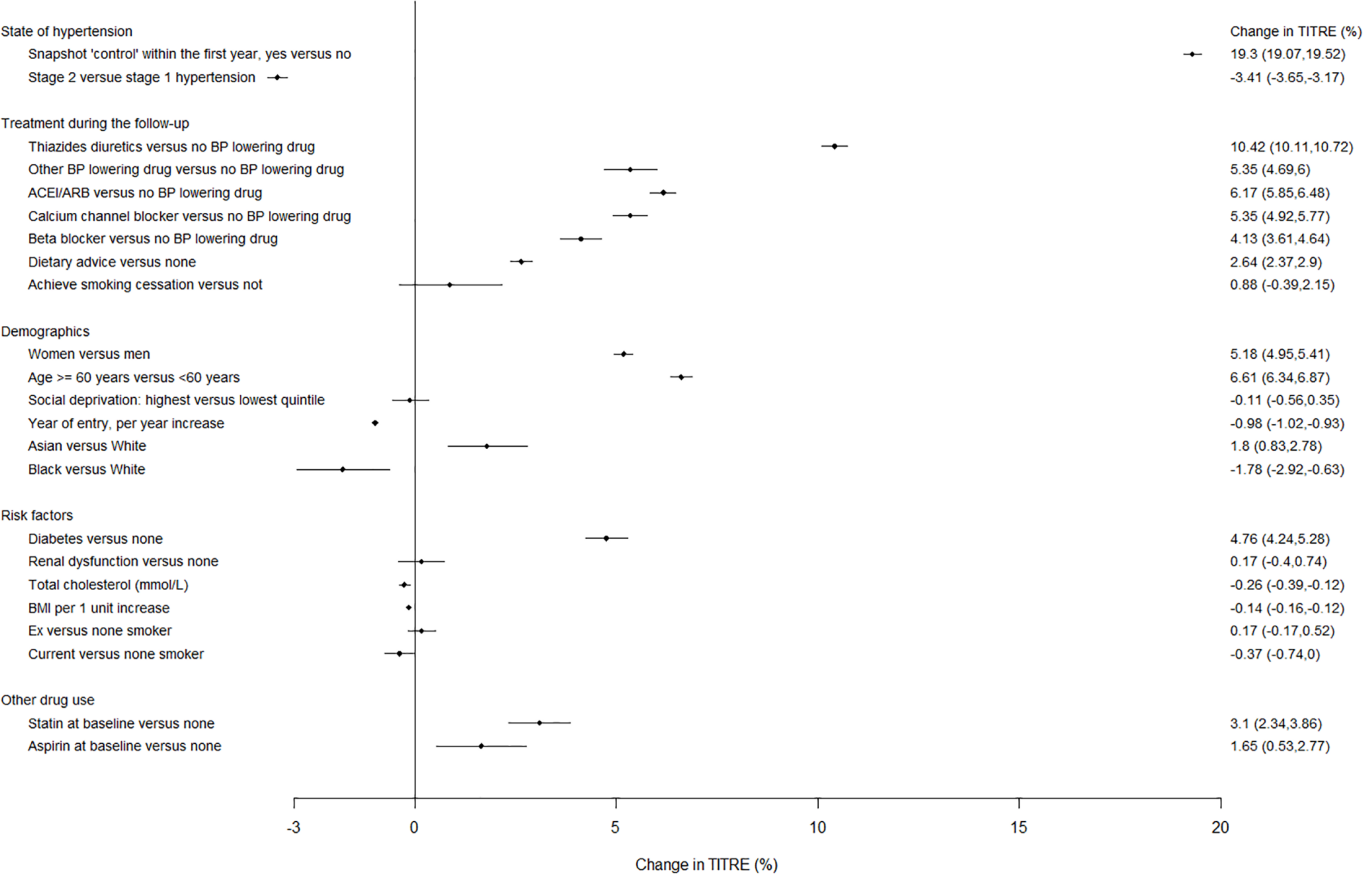
Patient characteristics and their association with a higher or lower TITRE (%).

We used a total of 1,640,460 follow-up BP readings (median, interquartile range of 7 (3, 16) readings per patient) to calculate TITRE. 25,866 (15.3%) of the 169,082 study population never reached control during follow-up; 51,819 (30.7%) patients had a TITRE less than three months, 39,651 (23.4%) three to six months, 25,237 (14.9%) six to nine months, and 7,557 (4.5%) nine to twelve months, and 18,952 (11.2%) had BP measurements unrecorded or more than one year apart during follow-up (Table 1). The frequency distribution of TITRE in the study population was positively skewed, with a median (first quartile, third quartile) of 23.5% (2.7%, 46.9%), which was equivalent to 2.8 (0.3, 5.6) months (Fig 2). Almost all individuals 99.4% (n = 168005) had a TITRE of less than 11 months per year. The strongest associations with higher TITRE categories was achieving snapshot ‘control’ during the first year; stage two hypertension showed negative associations with TITRE (Fig 3).

### Association between TITRE categories and mortality and cardiovascular endpoints

**Fig 4:**
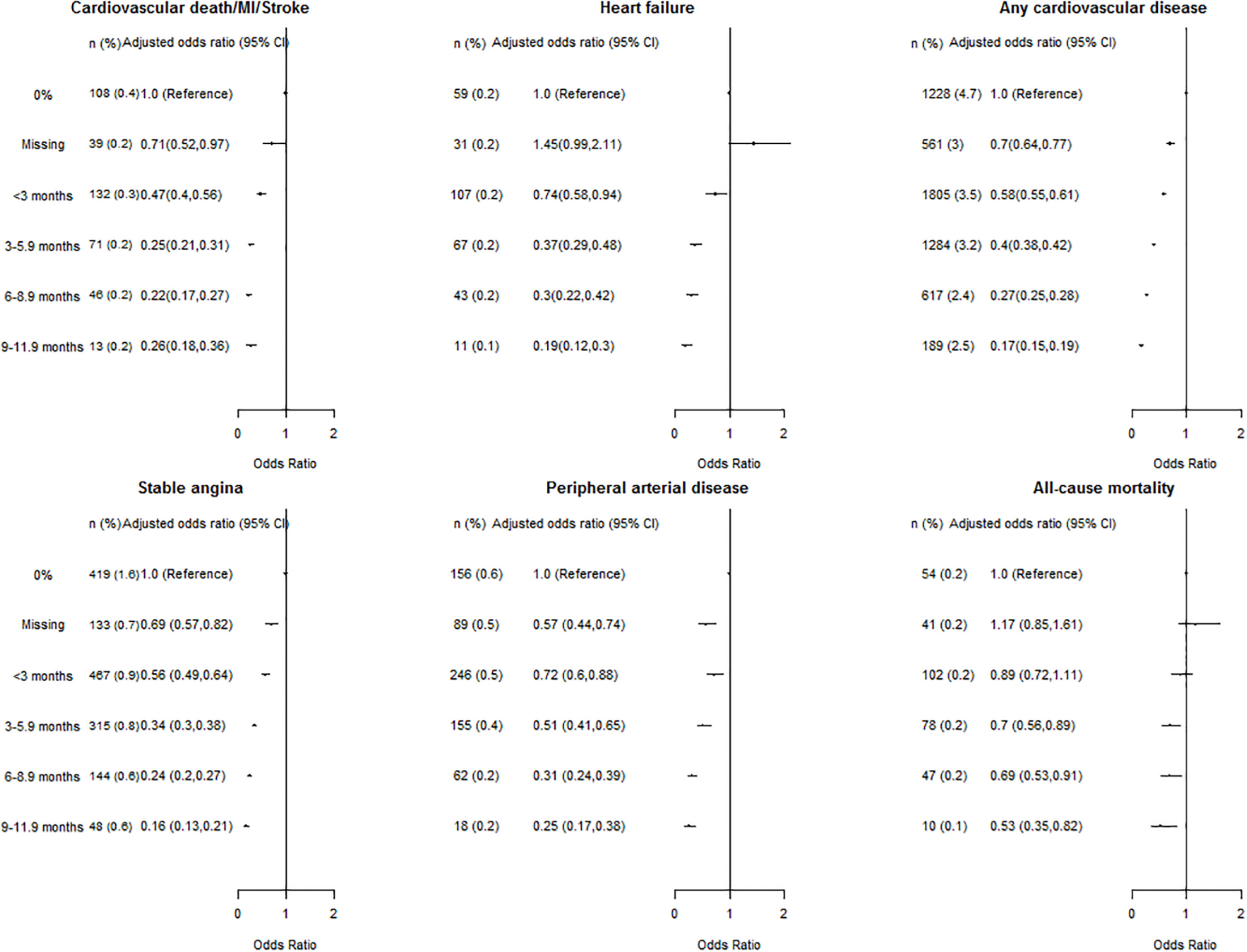
Associations between categorical time at target (TITRE) and primary (composite of cardiovascular mortality, myocardial infarction and stroke, incident heart failure and any cardiovascular diseases) and secondary (stable angina, peripheral artery disease, and all-cause mortality) study endpoints (n = 169082).

There was a graded, stepwise association between higher TITRE and lower risk of all outcomes. For the composite of cardiovascular death, myocardial infarction or stroke, by comparison with people with a 0% TITRE, individuals with a TITRE of <3 months, 3–5.9 months, 6–8.9 months, and 9–11.9 months had multiple adjusted odds ratio [OR] of 0.47 (95% confidence interval: 0.40 to 0.56), 0.25(0.21,0.31), 0.22(0.17,0.27) and 0.26(0.18,0.36), respectively (Fig 4). A higher TITRE was associated with lower risk of heart failure and any cardiovascular disease and death (odds ratio comparing 3–5.9 months, 6–8.9 months to 0% TITRE: 0.37 (0.29,0.48) and 0.30 (0.22,0.42) for heart failure; 0.40 (0.38,0.42) and 0.27 (0.25,0.28) for any cardiovascular diseases and death, respectively). In comparison, the association between TITRE and non-cardiovascular mortality was much weaker (TITRE 3–5.9 months compared to 0%: 0.74 (0.58,0.94)) to insignificant (TITRE <3 months compared to 0%: 0.98 (0.78,1.23), S1 Table), than the association between TITRE and cardiovascular events, suggesting that the observed association between higher TITRE and lower risk of cardiovascular outcomes was largely attributable to a well-maintained blood pressure level and not only to the general improvement of medical care only.

For secondary endpoints, the graded and stepwise associations were observed for TITRE and incident stable angina, peripheral artery disease, and all-cause mortality were also observed. For example, individuals with a TITRE of <3 months, 3–5.9 months, 6–8.9 months, and 9–11.9 months had multiple adjusted odds ratio [OR] of stable angina of 0.56 (0.49,0.64), 0.34 (0.3,0.38), 0.24 (0.2,0.27) and 0.16 (0.13,0.21), respectively. The risk of multicollinearity in study models was small, the area under the ROC curve of study models ranged from 0.81, 0.90, indicating good fit to the data, and Akaike’s information criteria suggested good prognostic value of TITRE (S2 Method).

In a hypothetical scenario, if patients experienced the standardized event risk of one level higher TITRE category than their own, in every 100,000 hypertensive individuals in the UK, 1824 deaths and cardiovascular disease events might be prevented or deferred over five years. In this hypothetical scenario, the greatest number of events prevented would be for patients with 0% or missing TITRE who, with TITRE of <3 months, would see an estimated 1990 deaths and cardiovascular diseases prevented or deferred, including 175 cardiovascular deaths, myocardial infarctions and strokes and 153 cases of incident heart failure (S3 Table).

### TITRE and other indicators of blood pressure control

Unadjusted event risks were similar in people who either achieved or did not achieve snapshot ‘control’ during the first year of follow-up (S4 Table). A consistent trend of stepwise relation between higher TITRE and risk of all outcomes was observed among patients who achieved or did not achieve a snapshot measure of blood pressure ‘control’ (S4 Table). Table 2 showed that binary control status was associated with a moderately lower risk of all cardiovascular events (0.78 (0.76, 0.81) for snapshot control during the first year of follow-up, and (0.73 (0.71, 0.76) for binary control defined by mean follow-up BP values). However, unlike TITRE, this single risk estimate failed to provide information on longitudinal blood pressure management status and its dose-response relationship with outcomes. For mean follow-up BP values, a 7.5 mmHg decrease in systolic blood pressure was associated with a 17% lower odds of any cardiovascular disease and death (adjusted odds ratio: 0.83 (0.83, 0.85)). The effect size was however lower than the 60% reduction in risk comparing TITRE of a current guideline recommended value of 3–6 months[6,31] to 0% (0.40 (0.38,0.42)) (Table 2). The use of systolic blood pressure variability in replacement of TITRE, showed close to null associations with outcomes. Additional adjustment for BP variability or number of follow-up measures did not change the stepwise relation between higher TITRE and cardiovascular endpoints.

**Table 2:** Categorical time at target (TITRE), alternative measures of BP control and any cardiovascular disease.

Measures of BP control	Adjusted odds ratio (95% CI)
**Binary**	All CVD/death
Snapshot ‘control’ yes vs. no^1^	0.78 (0.76,0.81)
Control’ yes vs. no, defined with mean follow-up systolic blood pressure^1^	0.73 (0.71, 0.76)
**Mean follow-up blood pressure**^1^	
Mean systolic blood pressure, per 7.5 mmHg decrease	0.83 (0.83, 0.85)
Mean diastolic blood pressure, per 5 mmHg decrease	0.93 (0.92, 0.94)
**Blood pressure variability**^1^^,^^2^	
Standard deviation of systolic blood pressure, per 1 unit decrease	0.98 (0.98, 0.98)
**TITRE**^1^	
0%	1.0 (Reference)
Missing	0.7 (0.64,0.77)
<3 months	0.58 (0.55,0.61)
3–5.9 months	0.40 (0.38,0.42)
6–8.9 months	0.27 (0.25,0.28)
9–11.9 months	0.17 (0.15,0.19)
**TITRE**^1^, **With additional adjustment of systolic BP variability**^2^	
0%	1.0 (Reference)
Missing	N/A
<3 months	0.57 (0.54,0.6)
3–5.9 months	0.40 (0.38,0.43)
6–8.9 months	0.27 (0.25,0.29)
9–11.9 months	0.18 (0.16,0.2)
**TITRE**^1^, **with additional adjustment of number of BP measures**	
0%	1.0 (Reference)
Missing	0.67 (0.62, 0.74)
<3 months	0.69 (0.65,0.72)
3–5.9 months	0.52 (0.49,0.55)
6–8.9 months	0.36 (0.34,0.38)
9–11.9 months	0.22 (0.2,0.25)

^1^Adjusted for age, gender, year of study entry, multiple deprivation, ethnicity, BMI, smoking, history of diabetes, renal dysfunction, stage two hypertension, total cholesterol, statin use, aspirin use, initial blood pressure lowing drug type, dietary advice, smoking cessation, snapshot ‘control’ status (except for models with binary BP control measures).

^2^blood pressure variability was measured by the standard deviation of follow-up blood pressure measures of each patient.

## Discussion

We report the first study investigating the time profiles of BP at target in usual care and subsequent risks of death and incident cardiovascular diseases. In 169,082 people with newly identified high BP, and 1.64 million BP measurements over a median of nearly five years, we estimated that BP was at current target levels for less than three months per year in half of all cases. Compared to a 0% TITRE, a TITRE based on current guideline recommendation (3–6 months)[6,31] was associated with a 75% reduction in odds of the composite of cardiovascular death, myocardial infarction and stroke, 63% reduction in incidence heart failure, and 60% reduction in the odds of any cardiovascular disease and death. The association was independent of the patients’ snapshot ‘BP control’ status within the first year and other BP management indicators. These findings suggest the need for more frequent measurement of BP in usual care and further research to understand how it may inform better blood pressure control.

### How often is blood pressure measured in usual care

A key finding from our study was how often blood pressure is measured in the 4.9 years after newly diagnosed hypertension. We found a median of 7 measurements per patient and this translated into a median of 1.6 measures per year. Clearly a more accurate measurement of TITRE would be made if BP measurements were more frequent; the new evidence we provide here is that even at this frequency of measurement it is possible to distinguish clinically relevant (in terms of prognosis) groups based on TITRE.

### Poor BP control: More critical when time course is considered

Clinical guidelines and many previous observational studies have defined ‘BP control’ as a binary variable, based on single cross-sectional BP measurement[8,35] above a given threshold. Studies using these definitions have found that 50%-81% of unselected populations[7–10,35] are not controlled (52.9% in the present study). Indeed, according to the totality of the primary care electronic health record only 0.5% of patients were observed to be controlled for an average of at least 11 months in a year); 15% of patients never had a recorded BP at target levels at any time point during follow-up. Though the true picture of longitudinal control based on a patient’s usual daily BP (which is not captured in usual practice) may be better than we observed, our findings are important as they utilize the complete BP information available to the treating physician.

### Factors associated with higher TITRE

A higher TITRE was associated with a number of factors, including; achieving BP control at a single time point within the year, and guideline-recommended intervention such as initial prescription of thiazides for BP lowering and provision of dietary advice. It is worth noting that, half of the hypertensive patients were not prescribed BP-lowering medications, suggesting substantial variation in hypertension management with usual care, combining lifestyle intervention, blood pressure monitoring and pharmaceutical intervention. The adherence to treatment strategies has also been shown to influence the management of blood pressure.[36] This contrasts with clinical trials, where almost all patients receive BP–lowering medication with regular clinical follow-up specified in trial protocols to enhance adherence,[3,12–14,19] even in usual care arms.[37] TITRE may help to assess the effectiveness of different hypertension management strategies.

### TITRE association with cardiovascular endpoints

A higher TITRE was associated with lower cardiovascular mortality and morbidity; furthermore, lower TITRE had a stronger association with risk of cardiovascular outcomes. The gradient of risk of cardiovascular diseases across different TITRE categories demonstrates not only its capability to better quantify the attributable risk to differences in longitudinal BP management, but also its potential to better characterize the benefits of BP-lowering interventions in reducing cardiovascular risk and mortality. The gradient of risk associated with TITRE remains after taking account of other BP management indicators, and in strata defined by antihypertensive drug use (S5 Table) and cross-sectional BP ‘control’ status (S4 Table). Sensitivity analyses showed that associations with cardiovascular endpoints and TITRE were predominantly consistent (S6 Table), and robust to the average number of follow-up BP measures a patient had. We present TITRE as an annual value, but its calculation is based on all measurements over follow-up which averaged 5 years. It thus quantifies the long-term pattern of blood pressure management.

It is worth noting that achieving a high TITRE at current BP targets was associated with relative risk reductions (and potential benefits) greater than reported in a recent meta-analysis of 19 randomized trials of intensive blood pressure lowering medication, which reported a 14% reduction in a composite of major cardiovascular events, but no clear effect on heart failure.[20] This contrasts with the 75% and 63% reductions in odds of major cardiovascular events and heart failure with a TITRE of 3–6 months compared to 0%. For clinical policy it may be more realistic and effective, to achieve higher TITRES at existing BP targets, than advocate even lower target BP levels such as those used in the recent SPRINT trial.[13]

Our study showed that the effects of TITRE on cardiovascular outcomes were greater and more informative than more commonly advocated measures of BP control. Thus snapshot measures cannot readily detect episodic hypertension in which systolic blood pressure oscillates above and below targets,[38] and commonly leads to premature cessation of anti-hypertensive treatment.[17] Mean follow-up blood pressure is commonly used as a summary measure of longitudinal blood pressure profile but fails to capture the cardiovascular risk of blood pressure surges, or to separate the effects of hypertensive and normotensive periods. Blood pressure variability captures the visit-to-visit blood pressure variation shown to be significantly associated with cardiovascular outcomes,[17,18] and has been incorporated into risk prediction algorithms.[39] Certainly, in the present study, snapshot and mean measures of blood pressure control were considerably weaker predictors of cardiovascular events than TITRE which effectively negated associations between blood pressure visit-to-visit variability and cardiovascular outcomes.

### Clinical implications

Clinicians and policymakers face choices between widely different strategies for managing BP. Our study suggests that before considering more aggressive treatment and lower BP targets, it might be more effective to focus on achieving, and maintaining BP control using existing blood pressure targets—a goal that is not achieved in US, Europe, or in low and middle income countries.[7–9,39–41] Countries with different antihypertension treatment strategies or medication prescription patterns may have a different TITRE profile in patients. We report an estimated 1823 cases of any cardiovascular disease and deaths that might be prevented per 100,000 newly diagnosed hypertensive patients over 5 years by increasing patients’ TITREs a level higher. Particular emphasis could be placed on facilitating patients with 0% or missing TITRE to achieve a TITRE of <3 months.

There is currently no mention in US,[4] European[2] or UK[6] guidelines of time at target for BP management. The updated guideline for high blood pressure management recommends a lower diastolic blood pressure target.[11] If improving TITRE cannot be achieved at higher target levels, it would seem even more challenging to expect clinicians, outside the context of trial settings, to deliver and sustain control at even lower BP target levels.[11] The Clinical guidelines could recommend the consideration of time at target as a readily implementable measure of the quality of care for this long-term condition; this may complement the current snapshot definition of control. Clinical precedents for such an approach come from consideration of time in therapeutic range for warfarin or using hemoglobin A1C as a marker of glycemic control over the preceding three months. Current clinical guidelines lack recommendations about the frequency of monitoring of hypertensive patients. Our findings support practice recommendations of more frequent measurement. A three-monthly monitoring interval is suggested with evidence from our findings of lower incremental cardiovascular risk with every three-month increase in TITRE, and previous research reported a more than 2.7 months delay in follow-up after intensification of BP-lowering treatment was associated with a greater risk of death and cardiovascular diseases.[42] Opportunities for monitoring every three-month are already present, as median number of consultations and visits in primary care was 4.7 per year per person population, where BP could be recorded during these visits in hypertensive patients and BP-lowering interventions initiated or titrated.

Implementation of the TITRE is feasible in electronic health records systems used to monitor clinical care and by patients themselves using home BP monitoring and apps. It could be further accompanied by visualizations of the time profile and decision support to target BP-lowering management.[43,44]

### Research implications—Need for trials of interventions to improve TITRE

Our findings suggest the need for international comparative study on TITRE and its association with cardiovascular risk in other health care system with different strategies in hypertension management (for example. different pattern of hypertensive medication use), and pragmatic real-world randomised trials evaluating interventions (e.g. decision support systems for clinician, patient or both) to increase the time spent at BP target, the best strategies to measure time at target (e.g. value of clinic versus self-measurement; frequency of measurement) and their impact on the incidence of cardiovascular diseases. In considering such trials and treatment guidelines, future research is required to evaluate whether strategies aimed at achieving a 20 mmHg lower BP target is more effective than strategies aimed at extending TITRE by 3 or more months.

The strengths of this study lie in this large nationally representative population from a country which incentivizes family physicians to meet the same national BP targets.[21] CALIBER’s linked electronic health records with repeated measures of clinically recorded BP among newly identified hypertension, and the large sample size provided unique opportunity for investigation of the TITRE and its association with major endpoints used in BP lowering trials. Our study has further limitations beyond confounding. TITRE may be less accurately defined in people with fewer recorded BP measures. Sensitivity analyses showed that associations with cardiovascular endpoints and TITRE were predominantly consistent across groups defined by number of follow-up BP measure categories (S6 Table). In studies of usual clinical care, it is not possible to control for error in blood pressure measurement arising from the lack of rigorous standardization. Nonetheless a degree of standardization is likely as a result of national clinical guidelines[29–31] coupled with financial incentives.[21] Our study had missing data (S7 Table) but we found consistent associations with cardiovascular events in imputed and complete case analyses (S8 Table).

## Conclusions

Almost all newly identified individuals with high BP fail to achieve and sustain consistent blood pressure control. Higher time at target (TITRE) was associated with lower risk of incident cardiovascular diseases and was an effective predictor of clinical outcomes, with a high clinical relevance and actionability. Efforts to lower cardiovascular risk among hypertensive patients should fully utilize multiple measurements of BP in usual care or via self-monitoring, with treatment strategies to attain a high TITRE.

## Supporting information

S1 Method Calculation of time at target (TITRE). (DOCX)

S2 Method Model examination and performance results. (DOCX)

S3 Method Estimated deaths and cardiovascular events delayed or deferred. (DOCX)

S4 Method Management of missing values. (DOCX)

S1 Fig Annual time at target blood pressure (%), by follow-up year and year of newly identified hypertension. (DOCX)

S1 Table Categorical time at target (TITRE) and risk of non-cardiovascular mortality. (DOCX)

S2 Table Akaike’s information criteria comparing performances of models for all cardiovascular diseases and death. (DOCX)

S3 Table Estimation of cardiovascular deaths and events delayed or prevented if BP control increases by one category of TITRE. (DOCX)

S4 Table Snapshot control status and risk of all cardiovascular disease and death according to time at target (TITRE). (DOCX)

S5 Table Antihypertensive medication use and risk of all cardiovascular disease and death according to time at target (TITRE) categories. (DOCX)

S6 Table Categorical time at target (TITRE) and risk of all cardiovascular disease and death by groups defined by average number of follow-up blood pressure measure categories. (DOCX)

S7 Table The extent of missing (%) of case-mix and treatment variables for multivariate models. (DOCX)

S8 Table Categorical time at target (TITRE) and risk of all cardiovascular disease and death in multiple imputed data and complete case analyses. (DOCX)
