# Supplementary material for: Time spent at blood pressure target and the risk of death and cardiovascular diseases
Source: PLoS One. 2018 Sep 5;13(9):e0202359. doi: 10.1371/journal.pone.0202359 (PMC6124703; doi:10.1371/journal.pone.0202359)
Supplement: S1 Method — (DOCX) [file pone.0202359.s001.docx]

**S1 method**: Calculation of time at target (TITRE).

The method begins with the calculation of days blood pressure at target between two consecutive blood pressure measurements.^[[1]](#footnote-1)^ The calculation is based on blood pressure taken before end of follow-up (the earliest occurrence of the following: first cardiovascular disease presentation, deregistration from the practice, final practice data collection, or end of the study).

**1. Four scenarios for every interval between any two consecutive blood pressure measures taken within one year defined by two anniversaries of study entry.**

Systolic or diastolic blood pressure (mmHg)

blood pressure target

At target days

At target days

At target days

blood pressure target

Days

Days

Days

Days

2

4

1

Scenario 1: interval in days classified as at target if measure in the beginning (lagbp) and at the end (bp) were both at target

Scenario 2: interval in days classified as not at target if measure in the beginning and at the end were both not at target

Scenario 3: from not at target (high blood pressure (lagbp)) to at target (low blood pressure (bp)), assuming linear change

If change occurred in systolic blood pressure (sbp);

Days at target=interval*((lagsbpcutoff-sbp)/(lagsbp-sbp))

If there was no systolic blood pressure change, and change occurred in diastolic blood pressure (dbp);

Days at target=interval*((lagdbpcutoff-dbp)/(lagdbp-dbp))

Scenario 4: from at target (low blood pressure (lagbp)) to not at target (high blood pressure (bp)), assuming linear change

If change occurred in sbp;

Days at target= interval*((lagsbpcutoff-lagsbp)/(sbp-lagsbp))

If there was no sbp change, and change occurred in dbp;

Days at target= interval*((lagdbpcutoff-lagdbp)/(dbp-lagdbp))

**2. Scenarios for interval between two blood pressure measures that include follow-up anniversary**

Yr2 at target days

Yr1 at target days

blood pressure control cut-off

blood pressure control cut-off

Yr2 at target days

Yr1 at target days

Yr1 at target days

Yr2 at target days

1

2

3

4

*1^st^ anniversary from study entry*

*1^st^ anniversary from study entry*

Scenario 1: interval classified as at target days if measure in the beginning and at the end were both at target;

Days at target in year 1: D=(365.25)-lagdate;

Days at target in year 2: D=date-(365.25);

Scenario 2: interval classified as not at target days if measure in the beginning and at the end were both not at target; (no at target days)

Scenario 3: from not at target (high blood pressure (lagbp)) to in-control to low blood pressure (bp)

If change occurred in sbp;

Days at target=interval*((lagsbpcutoff-sbp)/(lagsbp-sbp))

If there was no sbp change, and change occurred in dbp;

Days at target=interval*((lagdbpcutoff-dbp)/(lagdbp-dbp))

Find out how many days in-control days were in year 1 and how many days were in year 2

x=date-(days at target)

if x>=365.25 then yr2 at target days= days at target; yr1 at target days =0

if x<365.25 then yr1 at target days=365.25-x; yr2 at target days =date-365.25

Scenario 4: from at target to not at target (low blood pressure (lagbp, lagbpcutoff) to high bp (bp)

If change occurred in sbp;

Days at target= interval*((lagsbpcutoff-lagsbp)/(sbp-lagsbp))

If there was no sbp change, and change occurred in dbp;

Days at target= interval*((lagdbpcutoff-lagdbp)/(dbp-lagdbp))

Find out how many days at target days were in year 1 and how many days were in year 2

x=lagdate + (days at target)

if x>=365.25 then yr1 at target days=365.25-lagdate; yr2 at target days=x-365.25.

if x<365.25 then yr1 at target days= at target days; yr2 at target days=0.

**3.** For each patient, sum up days at target for blood pressure measures taken during each follow-up year;

**4.** Then find the days the patient was followed in the follow-up year to calculate percent time at target for each follow-up year;

**5.** For each follow-up year, the days when BP was at treatment target between consecutive records were then summed to calculate the annual percent time at target. (Figure S1) Measures recorded more than one year from the previous recorded BP values were not included in the calculation.

**6.** Finally obtain the mean over follow-up years as the average time at target (TITRE) for each patient.

Example: Calculating time at target (TITRE) for a hypothetical patient

*Blood pressure measures taken over time for the patient:*

1^st^ anniversary

2^nd^ anniversary

3^rd^ anniversary

4^th^ anniversary

Yr1 at target days

Yr2 at target days

Yr4 at target days

Yr5 at target days

Study entry (enter at the latest baseline high blood pressure measure date)

Study exit (3 month after 4^th^ anniversary)

blood pressure control cut-off

Assume the patient’s at targets days were 150 in year one, 210 in year two, 0 in year three, 190 in year four and 90 in year five.

The yearly time at target would be:

Year one: 150/365.25 = 41%

Year two: 210/365.25 = 57%

Year three: 0/365.25 = 0%

Year four: 190/365.25 = 52%

Year five: 90/90 = 100%

The time at target (TITRE) would be (0.41+0.57+0+0.52+1)/5=50

**7.** The first, second and third quartiles of TITRE were 2.7% (0.3 months), 23.5% (2.8 months), and 46.9% (5.6 months). As the difference between quartiles was about 3 months, we chose a 3-month interval for TITRE categories. People who never had a follow-up BP record after the high blood pressure diagnosis, and hypertensive patients who had never reached target are of clinical importance. We thus categorized TITRE into six groups: missing, 0 time, less than 3 months, 3 to 5.9 months, 6 to 8.9 months, and 9 to 11.9 months.

Example SAS macro for TITRE calculation

d_entry_to_bp: days from cohort entry to a blood pressure measures;

dob: date of birth;

bpdate: date of a blood pressure measure;

sys_bp: systolic blood pressure of a measure;

dias_bp: diastolic blood pressure of a measure;

diab_base: history of diabetes at study entry;

hist_renal: history of chronic kidney disease at study entry;

date_entry: date of study entry;

*Identify the follow-up year where each eligible BP was measured;

data sample; set sample;

do i=1 to 12;

if d_entry_to_bp>=365.25*i and d_entry_to_bp < 365.25*(i+1) then yearly=(i+1);

end;

if d_entry_to_bp>=0 and d_entry_to_bp < 365.25 then yearly=1;

run;

*Find out in control and not in-control bp measures (sys and dias seperately) based on the patient condition at the time of measurement;

data sample; set sample;

bd60=dob+365.25*60;

format bd60 mmddyy10.;

if missing(bpdate) eq 0 and missing(sys_bp) eq 0 and missing(dias_bp) eq 0 then do;

incontrol=((sys_bp<140) and (dias_bp<90));

sysincontrol=(sys_bp<140);

diasincontrol=(dias_bp<90);

sbpcutoff=139.99;diascutoff=89.99;

if (bpdate ge bd60) and (diab_base eq 0) and missing(hist_renal) then do;

incontrol=((sys_bp<150) and (dias_bp<90));

sysincontrol=(sys_bp<150);

diasincontrol=(dias_bp<90);

sbpcutoff=149.99;diascutoff=89.99;

end;

if missing (hist_renal) eq 0 then do;

if (bpdate ge bd60) and (diab_base eq 0) and (bpdate < hist_renal) then do;

incontrol=((sys_bp<150) and (dias_bp<90));

sysincontrol=(sys_bp<150);

diasincontrol=(dias_bp<90);

sbpcutoff=149.99;diascutoff=89.99;

end;

end;

end;

*Baseline control status and BP cutoff;

if missing(date_entry) eq 0 and missing(sbp_at_entry) eq 0 and missing(dbp_at_entry) eq 0 then do;

baseline_incontrol=((sbp_at_entry<140) and (dbp_at_entry<90));

baseline_sysincontrol=(sbp_at_entry<140);

baseline_diasincontrol=(dbp_at_entry<90);

baseline_sbpcutoff=139.99;baseline_diascutoff=89.99;

if (date_entry ge bd60) and (diab_base eq 0) and missing(hist_renal) then do;

baseline_incontrol=((sbp_at_entry<150) and (dbp_at_entry<90));

baseline_sysincontrol=(sbp_at_entry<150);

baseline_diasincontrol=(dbp_at_entry<90);

baseline_sbpcutoff=149.99;baseline_diascutoff=89.99;

end;

if missing (hist_renal) eq 0 then do;

if (date_entry ge bd60) and (diab_base eq 0) and (date_entry < hist_renal) then do;

baseline_incontrol=((sbp_at_entry<150) and (dbp_at_entry<90));

baseline_sysincontrol=(sbp_at_entry<150);

baseline_diasincontrol=(dbp_at_entry<90);

baseline_sbpcutoff=149.99;baseline_diascutoff=89.99;

end;

end;

end;

run;

data sample; set sample;

lagsbp=lag(sys_bp);

if id ne lag(id) then lagsbp=sbp_at_entry;

lagdbp=lag(dias_bp);

if id ne lag(id) then lagdbp=dbp_at_entry;

lagsbpcutoff=lag(sbpcutoff);

if id ne lag(id) then lagsbpcutoff=baseline_sbpcutoff;

lagdiascutoff=lag(diascutoff);

if id ne lag(id) then lagdiascutoff=baseline_diascutoff;

*Measure with > 1 year interval not included in the % time calculation with the previous measure (but can be included if the interval with the subsequent measure was less than one year apart);

d_from_previous_bp_1=d_from_previous_bp; if d_from_previous_bp_gt1yr then d_from_previous_bp_1=.;

interval=d_from_previous_bp_1;

if missing(interval) then lagsbp=.;

if missing(interval) then lagdbp=.;

if missing(interval) then lagsbpcutoff=.;

if missing(interval) then lagdiascutoff=.;

lagincontrol=lag(incontrol);

if id ne lag(id) then lagincontrol=baseline_incontrol;

lagsysincontrol=lag(sysincontrol);

if id ne lag(id) then lagsysincontrol=baseline_sysincontrol;

lagdiasincontrol=lag(diasincontrol);

if id ne lag(id) then lagdiasincontrol=baseline_diasincontrol;

lag_d_entry_to_bp=lag(d_entry_to_bp);

lagyearly=lag(yearly);

if id ne lag(id) then lagyearly=0;

run;

*Now begin to calculate TITRE;

%macro *OnTargetDay*;

data OnTargetDay; set sample;

%do i=1 %to 13;

%let j = %Eval( &i + 1 ) ;

%let k = %Eval( &i - 1 ) ;

%let m = %sysevalf(365.25*&i) ;

if (yearly eq &i.) and (lagyearly in (&i., &k.)) and missing(yr&i.incontrold) then do;

if incontrol eq 1 and lagincontrol eq 1 then yr&i.incontrold=interval;

if incontrol eq 0 and lagincontrol eq 0 then yr&i.incontrold=0;

if incontrol eq 1 and lagincontrol eq 0 then do;

if (sysincontrol eq 1) and (lagsysincontrol eq 0) then do;

if (lagsbp ne sys_bp) then yr&i.incontrold=interval*((lagsbpcutoff-sys_bp)/(lagsbp-sys_bp));

end;

if ((sysincontrol=lagsysincontrol) and ((diasincontrol eq 1) and (lagdiasincontrol eq 0)))

or ((sysincontrol eq 1) and (lagsysincontrol eq 0) and (lagsbp eq sys_bp))

then do;

if (lagdbp ne dias_bp) then yr&i.incontrold=interval*((lagdiascutoff-dias_bp)/(lagdbp-dias_bp));

end;

end;

if incontrol eq 0 and lagincontrol eq 1 then do;

if (sysincontrol eq 0) and (lagsysincontrol eq 1) then do;

if (lagsbp ne sys_bp) then yr&i.incontrold=interval*((lagsbpcutoff-lagsbp)/(sys_bp-lagsbp));

end;

if ((sysincontrol=lagsysincontrol) and ((diasincontrol eq 0) and (lagdiasincontrol eq 1)))

or ((sysincontrol eq 0) and (lagsysincontrol eq 1) and (lagsbp eq sys_bp))

then do;

if (lagdbp-dias_bp) then yr&i.incontrold=interval*((lagdiascutoff-lagdbp)/(dias_bp-lagdbp));

end;

end;

end;

if missing(interval) eq 0 and ((yearly eq &j. and lagyearly eq &i.)) then do;

if incontrol eq 1 and lagincontrol eq 1 then do;

yr&i.incontrold=&m.-lag_d_entry_to_bp;

yr&j.incontrold=d_entry_to_bp-&m.;

end;

if incontrol eq 0 and lagincontrol eq 0 then do; yr&i.incontrold=0; yr&j.incontrold=0;end;

if incontrol eq 1 and lagincontrol eq 0 then do;

if (sysincontrol eq 1) and (lagsysincontrol eq 0) then do;

if (lagsbp ne sys_bp) then incontrold=interval*((lagsbpcutoff-sys_bp)/(lagsbp-sys_bp));

end;

if ((sysincontrol=lagsysincontrol) and ((diasincontrol eq 1) and (lagdiasincontrol eq 0)))

or ((sysincontrol eq 1) and (lagsysincontrol eq 0) and (lagsbp eq sys_bp))

then do;

if (lagdbp ne dias_bp) then incontrold=interval*((lagdiascutoff-dias_bp)/(lagdbp-dias_bp));

end;

x=d_entry_to_bp-incontrold;

if x>=&m. then do; yr&j.incontrold=incontrold; yr&i.incontrold=0;end;

if x<&m. then do; yr&i.incontrold=&m.-x; yr&j.incontrold =d_entry_to_bp-&m.;end;

end;

if incontrol eq 0 and lagincontrol eq 1 then do;

if (sysincontrol eq 0) and (lagsysincontrol eq 1) then do;

if (lagsbp ne sys_bp) then incontrold=interval*((lagsbpcutoff-lagsbp)/(sys_bp-lagsbp));

end;

if ((sysincontrol=lagsysincontrol) and ((diasincontrol eq 0) and (lagdiasincontrol eq 1)))

or ((sysincontrol eq 0) and (lagsysincontrol eq 1) and (lagsbp eq sys_bp))

then do;

if (lagdbp ne dias_bp) then incontrold=interval*((lagdiascutoff-lagdbp)/(dias_bp-lagdbp));

end;

x=lag_d_entry_to_bp+incontrold;

if x>=&m. then do; yr&i.incontrold=&m.-lag_d_entry_to_bp; yr&j.incontrold=x-&m.;end;

if x<&m. then do; yr&i.incontrold=incontrold; yr&j.incontrold=0;end;

end;

end;

if missing(yr&i.incontrold) eq 0 and (sbpcutoff ne lagsbpcutoff) then do;

yr&i.incontrold=.;

end;

if missing(yr&j.incontrold) eq 0 and (sbpcutoff ne lagsbpcutoff) then do;

yr&j.incontrold=.;

end;

%end;

run;

%mend;

%*OnTargetDay*;

proc sql;

create table yearly as

select unique(id),

sum (yr1incontrold) as sumyr1incontrold,

sum (yr2incontrold) as sumyr2incontrold,

sum (yr3incontrold) as sumyr3incontrold,

sum (yr4incontrold) as sumyr4incontrold,

sum (yr5incontrold) as sumyr5incontrold,

sum (yr6incontrold) as sumyr6incontrold,

sum (yr7incontrold) as sumyr7incontrold,

sum (yr8incontrold) as sumyr8incontrold,

sum (yr9incontrold) as sumyr9incontrold,

sum (yr10incontrold) as sumyr10incontrold,

sum (yr11incontrold) as sumyr11incontrold,

sum (yr12incontrold) as sumyr12incontrold,

sum (yr13incontrold) as sumyr13incontrold

from OnTargetDay

group by id;

quit;

proc sort data=sample; by id;

data sample1; set sample (keep=id date_entry date_exit_dx); by id; if first.id then output; run;

proc sort data=sample1; by id;

proc sort data=yearly; by id;

data TITRE; merge sample1 yearly; by id;run;

%macro *TITRE*;

data TITRE; set TITRE;

if date_exit_dx ge date_entry+365.25 then yr1_percent_incontrol=100*(sumyr1incontrold/365.25);

if date_exit_dx lt date_entry+365.25 then yr1_percent_incontrol=100*(sumyr1incontrold/(date_exit_dx-date_entry));

%do i=1 %to 12;

%let j = %Eval( &i + 1 ) ;

if date_exit_dx ge date_entry+%sysevalf(365.25*&i) and date_exit_dx ge date_entry+%sysevalf(365.25*(&i+1)) then yr&j._percent_incontrol=100*(sumyr&j.incontrold/365.25);

if date_exit_dx ge date_entry+%sysevalf(365.25*&i) and date_exit_dx lt date_entry+%sysevalf(365.25*(&i+1))then yr&j._percent_incontrol=100*(sumyr&j.incontrold/(date_exit_dx-%sysevalf(365.25*&i)));

%end;

run;

%mend;

%*TITRE*

*TITRE, averaged yearly % time in control;

data TITRE; set TITRE;

TITRE=mean(of yr1_percent_incontrol, yr2_percent_incontrol, yr3_percent_incontrol,yr4_percent_incontrol, yr5_percent_incontrol, yr6_percent_incontrol, yr7_percent_incontrol,yr8_percent_incontrol, yr9_percent_incontrol

, yr10_percent_incontrol, yr11_percent_incontrol,yr12_percent_incontrol, yr13_percent_incontrol);

*Categorical TITRE;

if TITRE EQ 0 then cTITRE=0;

if missing(TITRE)then cTITRE=1;

if TITRE>0 and TITRE <25 then cTITRE=2;

if TITRE>=25 and TITRE <50 then cTITRE=3;

if TITRE>=50 and TITRE <75 then cTITRE=4;

if TITRE>=75 then cTITRE=5;

run;

1. Please consult figure 1 of the study for the inclusion and exclusion criteria for blood pressure measures used for the calculation. [↑](#footnote-ref-1)
