# Supplementary material for: Time spent at blood pressure target and the risk of death and cardiovascular diseases
Source: PLoS One. 2018 Sep 5;13(9):e0202359. doi: 10.1371/journal.pone.0202359 (PMC6124703; doi:10.1371/journal.pone.0202359)
Supplement: S2 Method — (DOCX) [file pone.0202359.s002.docx]

**S2 method:** Model examination and performance results

The risk of multicollinearity in our models was small, as the condition indexes from collinearity diagnostics were low (ranging from 1.0 to 4.1), indicating TITRE categories and snapshot control status were fairly independent in their association with study endpoints. The area under the ROC curve was 0.83 from the models for the composite endpoint of cardiovascular death, MI, and stroke, 0.90 for incident heart failure and 0.81 for all cardiovascular disease, indicating good fit to the data.

To evaluate the prognostic power of TITRE, we performed the goodness of fit tests comparing the performance of the model of TITRE + mean SBP + covariates to the model of mean SBP + covariates. The comparison was based on the Akaike’s information criteria (AIC). The smaller AIC value indicates that model of TITRE + mean SBP + covariates performs better than the model of mean SBP + covariates, suggesting TITRE provide additional prognostic power compared with average BP only.
