# Supplementary material for: Time spent at blood pressure target and the risk of death and cardiovascular diseases
Source: PLoS One. 2018 Sep 5;13(9):e0202359. doi: 10.1371/journal.pone.0202359 (PMC6124703; doi:10.1371/journal.pone.0202359)
Supplement: S3 Method — (DOCX) [file pone.0202359.s003.docx]

**S3 method:** estimated deaths and cardiovascular events delayed or deferred

We evaluated the number of estimated deaths and cardiovascular events delayed or deferred, under the hypothetic scenario that patients experienced the standardized event risk of one level higher TITRE category than their own. The case mix standardized event risk was obtained by indirect standardization. The predicted probability of primary study endpoints for each patient was derived from generalized logistic models with case mix and treatment covariates. The individual patient probability was summarized by TITRE category as the expected event risk. The standardized risk ratio was obtained by dividing the observed by the expected event risk, and case mix standardized event risks were derived by multiplying each TITRE stratum-specific standardized risk ratio by the crude event risk of the respective endpoint in the whole study population.

Table S3 summarized the case mix and treatment standardized event risk by TITRE category, and estimated deaths and cardiovascular events delayed or prevented if patients experienced the standardized event risk of one level higher TITRE category than their own. Specifically, patients of 0% or missing TITRE had a standardized event risk for cardiovascular diseases in TITRE stratum of <3 months. Likewise, patients with TITRE categories of <3 months, 3 to 6 months and 6 to 9 months were assumed to experience the standardized event risk of cardiovascular diseases in TITRE stratum of 3 to 6 months, 6 to 9 months and more than 9 months, respectively. The greatest mean difference in risk-standardized mortality observed between adjacent TITRE groups was between 0% to <3 months in the composite of cardiovascular death, myocardial infarction and stroke (0.5%), missing to < 3 months in incident heart failure (0.5%), and 0% to < 3 months in all cardiovascular diseases (4.5%).

A TITRE increase of three months resulted in an estimated 3083 cardiovascular diseases prevented or deferred in the UK, 263 cases of cardiovascular deaths, myocardial infarction and stroke, and 237 incident heart failure. The greatest number of events prevented between adjacent TITRE categories was between 0% or missing to <3 months in cardiovascular death, preventing in total 175 events of myocardial infarction and stroke, 153 cases of incident heart failure, and 1990 of cardiovascular diseases. The expected number of cardiovascular events prevented or deferred per 100,000 patients with newly diagnosed hypertension over 5 years was 1824 deaths and cardiovascular diseases, 156 cardiovascular deaths, myocardial infarctions and strokes and 141 cases of incident heart failure per 100,000 hypertensive population over five years.
