# Supplementary material for: Time spent at blood pressure target and the risk of death and cardiovascular diseases
Source: PLoS One. 2018 Sep 5;13(9):e0202359. doi: 10.1371/journal.pone.0202359 (PMC6124703; doi:10.1371/journal.pone.0202359)
Supplement: S4 Method — (DOCX) [file pone.0202359.s004.docx]

**S4 method:** management of missing values

The extent of missing data is shown in Table S7. To appropriately utilize all available data specifically for the case-mix model of various cardiovascular diseases, we imputed missing case-mix variables using the SAS multiple imputation procedures. The procedure is following Rubin’s rule, replacing each missing value with a set of values which represent a random sample of the missing values.^1^ We used the Markov Chain Monte Carlo algorithm for arbitrary missing data patterns.^2^ The number of imputations in the study is 10.

In multiple imputation for missing case-mix information, we include case-mix variables (year of study entry, gender, index of multiple deprivation, race/ethnicity, history of smoking, height, weight, age at study entry, systolic blood pressure and diastolic blood pressure at study entry, baseline hemoglobin, while blood cell count, creatinine, total cholesterol, high density lipoprotein, pulse, albumin, estimated glomerular filtration rate, history or liver disease, chronic obstructive pulmonary disease, depression, anxiety, stage two hypertension, diabetes, and renal dysfunction), medication prior to study entry (statins use, aspirin use). For analyses based on multiple imputed data, imputed values for categorical variables were not rounded to avoid bias in the estimates.^3;4^

For sensitivity analyses to verify the results based on multiple imputed data, we construct case-mix models with study covariates with complete data (age, gender, year of study entry, multiple deprivation, ethnicity, BMI, smoking, history of diabetes, renal dysfunction, stage two hypertension, total cholesterol, statin use, aspirin use, initial blood pressure lowing drug type, dietary advice, smoking cessation, snapshot ‘control’ status for the top panel), where missing category was created for missing values in multiple deprivation, ethnicity, BMI, and smoking. The risk estimates for study endpoints from models with complete data were similar to estimates from imputed data (Table S8).

**Reference**

(1) Rubin DB. *Multiple imputation for nonresponse in surveys*. New York: Wiley, 1987.

(2) Yuan YC. Multiple Imputation for Missing Data: Concepts and New Development. 1-13. 2000. Twenty-Fifth Annual SAS Users Group International Conference. Cary, NC, SAS Institute.

(3) Allison PD. Imputation of categorical variables with PROC MI. 2005. The 30th Annual SAS Users Group International Conference (SUGI 30). Philadelphia, PA.

(4) Ake CF. Rounding after multiple imputation with non-binary categorical covariates. 2005. The 30th Annual SAS Users Group International Conference (SUGI 30). Philadelphia, PA.
