# Supplementary material for: Time spent at blood pressure target and the risk of death and cardiovascular diseases
Source: PLoS One. 2018 Sep 5;13(9):e0202359. doi: 10.1371/journal.pone.0202359 (PMC6124703; doi:10.1371/journal.pone.0202359)
Supplement: S1 Fig — (DOCX) [file pone.0202359.s005.docx]

**S1 Figure:** Annual time at target blood pressure (%), by follow-up year and year of newly identified hypertension. *p value all <0.0001 for nonparametric Kruskal-Wallis test comparing annual time at target (%) by year of entry.

Figure S1 showed that the annual time at target blood pressure increased over follow-up years (26.3% at the first year and 39.0% at 5^th^ year of follow-up). The annual time at target blood pressure also increased with later year of study entry (p for trend <0.0001).
