# Supplementary material for: Time spent at blood pressure target and the risk of death and cardiovascular diseases
Source: PLoS One. 2018 Sep 5;13(9):e0202359. doi: 10.1371/journal.pone.0202359 (PMC6124703; doi:10.1371/journal.pone.0202359)
Supplement: S1 Table — (DOCX) [file pone.0202359.s006.docx]

**S1 Table:** Categorical time at target (TITRE) and risk of non-cardiovascular mortality.

| TITRE category | Non-CVD mortality |
| --- | --- |
| 0% | 1.0 (Reference) |
| Missing | 1.24 (0.91,1.69) |
| <3 months | 0.98 (0.78,1.23) |
| 3-5.9 months | 0.74 (0.58,0.94) |
| 6-8.9 months | 0.75 (0.57,0.99) |
| 9-11.9 months | 0.51 (0.33,0.78) |
