# Supplementary material for: Time spent at blood pressure target and the risk of death and cardiovascular diseases
Source: PLoS One. 2018 Sep 5;13(9):e0202359. doi: 10.1371/journal.pone.0202359 (PMC6124703; doi:10.1371/journal.pone.0202359)
Supplement: S2 Table — (DOCX) [file pone.0202359.s007.docx]

**S2 Table:** Akaike’s information criteria comparing performances of models for all cardiovascular diseases and death.

|  | Covariates only model | Covariates + average DBP (per 5 mmHg) | Covariates + average SBP (per 7.5 mmHg) | Covariates + average SBP (per 7.5 mmHg) + TITRE (categorical variables) |
| --- | --- | --- | --- | --- |
| AIC (smaller is better) | 165340.8 | 159053.9 | 158334.9 | 156866.5 |
