# Supplementary material for: Time spent at blood pressure target and the risk of death and cardiovascular diseases
Source: PLoS One. 2018 Sep 5;13(9):e0202359. doi: 10.1371/journal.pone.0202359 (PMC6124703; doi:10.1371/journal.pone.0202359)
Supplement: S3 Table — (DOCX) [file pone.0202359.s008.docx]

**S3 Table:** Estimation of cardiovascular deaths and events delayed or prevented if BP control increases by one category of TITRE.

| Outcomes | Number of patients in TITRE groups | Number  of events | Observed  risk | Expected risk^1^ | SRR^2^ | Standardized event risk^3^ | Hypothetical increase in TITRE category | Expected number of event prevented | Expected number of cardiovascular events prevented per 100,000 patients with newly diagnosed hypertension over 5 years |
| --- | --- | --- | --- | --- | --- | --- | --- | --- | --- |
| **CV death /MI/Stroke** | |  |  |  |  |  |  |  |  |
| 0% | 25866 | 108 | 0.42% | 0.11% | 3.8 | 0.9% | 0% to <3 months | 122.6 |  |
| Missing | 18952 | 39 | 0.21% | 0.07% | 3.0 | 0.7% | missing to <3 months | 52.0 |  |
| <3 months | 51819 | 132 | 0.25% | 0.14% | 1.8 | 0.4% | <3 to 3-5.9 months | 95.4 |  |
| 3-5.9 months | 39651 | 71 | 0.18% | 0.17% | 1.1 | 0.3% | 3-5.9 to 6-8.9 months | 0.3 |  |
| 6-8.9 months | 25237 | 46 | 0.18% | 0.17% | 1.1 | 0.3% | 6-8.9 to 9-11.9 months | -7.8 |  |
| 9-11.9 months | 7557 | 13 | 0.17% | 0.14% | 1.2 | 0.3% | -- |  |  |
| Total | 169082 | 409 | 0.24% |  |  |  |  |  | 156 |
| **Heart Failure** | |  |  |  |  |  |  |  |  |
| 0% | 25866 | 59 | 0.23% | 0.07% | 3.2 | 0.6% | 0% to <3 months | 67.9 |  |
| Missing | 18952 | 31 | 0.16% | 0.04% | 4.1 | 0.8% | missing to <3 months | 84.5 |  |
| <3 months | 51819 | 107 | 0.21% | 0.12% | 1.8 | 0.3% | <3 to 3-5.9 months | 73.8 |  |
| 3-5.9 months | 39651 | 67 | 0.17% | 0.17% | 1.0 | 0.2% | 3-5.9 to 6-8.9 months | 11.3 |  |
| 6-8.9 months | 25237 | 43 | 0.17% | 0.20% | 0.9 | 0.2% | 6-8.9 to 9-11.9 months | -0.7 |  |
| 9-11.9 months | 7557 | 11 | 0.15% | 0.16% | 0.9 | 0.2% | -- |  |  |
| Total | 169082 | 318 | 0.19% |  |  |  |  |  | 141 |
| **All cardiovascular diseases and death** | | | |  |  |  |  |  |  |
| 0% | 25866 | 1228 | 4.75% | 1.54% | 3.1 | 10.3% | 0% to <3 months | 1177.9 |  |
| Missing | 18952 | 561 | 2.96% | 0.99% | 3.0 | 10.1% | missing to <3 months | 812.0 |  |
| <3 months | 51819 | 1805 | 3.48% | 2.02% | 1.7 | 5.8% | <3 to 3-5.9 months | 766.7 |  |
| 3-5.9 months | 39651 | 1284 | 3.24% | 2.53% | 1.3 | 4.3% | 3-5.9 to 6-8.9 months | 473.4 |  |
| 6-8.9 months | 25237 | 617 | 2.44% | 2.64% | 0.9 | 3.1% | 6-8.9 to 9-11.9 months | -147.2 |  |
| 9-11.9 months | 7557 | 189 | 2.50% | 2.27% | 1.1 | 3.7% | -- |  |  |
| Total | 169082 | 5684 | 3.4% |  |  |  |  |  | 1824 |

^1^Estimated based on generalized mixed model including case mix and treatment covariates. ^2^Standared risk ratio, generated by dividing observed by expected risk. ^3^Generated by multiply SRR by observed risk of the total population.
