# Supplementary material for: Time spent at blood pressure target and the risk of death and cardiovascular diseases
Source: PLoS One. 2018 Sep 5;13(9):e0202359. doi: 10.1371/journal.pone.0202359 (PMC6124703; doi:10.1371/journal.pone.0202359)
Supplement: S4 Table — (DOCX) [file pone.0202359.s009.docx]

**S4 Table:** Snapshot control status and risk of all cardiovascular disease and death according to time at target (TITRE)^1^.

| Adjusted odds ratio (95% CI) | | | |
| --- | --- | --- | --- |
|  | **Original results, all patients (n=169082)** | **Snapshot in control (n=79713)** | **Snapshot not in control (n=89369)** |
|  |  | N (%) : 2446 (3.1) | N (%) : 3238 (3.6) |
| 0% | 2.57 (2.43,2.71) | N/A | 2.91 (2.73,3.1) |
| Missing | 1.73 (1.58,1.89) | N/A | 2.16 (1.96,2.38) |
| <3 months | 1.46 (1.41,1.51) | 1.3 (1.23,1.38) | 1.61 (1.53,1.7) |
| 3-5.9 months | 1.0 (Reference) | 1.0 (Reference) | 1.0 (Reference) |
| 6-8.9 months | 0.67 (0.63,0.7) | 0.64 (0.6,0.68) | 0.7 (0.65,0.76) |
| 9-11.9 months | 0.42 (0.38,0.46) | 0.42 (0.38,0.46) | 0.33 (0.24,0.46) |

^1^Since 0% time is very few for patients achieved snapshot in control, the reference group of the comparison is set to the TITRE category of 3-5.9 months. The model is adjusted for age, gender, year of study entry, multiple deprivation, ethnicity, BMI, smoking, history of diabetes, renal dysfunction, stage two hypertension, total cholesterol, statin use, aspirin use, initial blood pressure lowing drug type, dietary advice, smoking cessation, snapshot ‘control’ status for the top panel.
