# Supplementary material for: Time spent at blood pressure target and the risk of death and cardiovascular diseases
Source: PLoS One. 2018 Sep 5;13(9):e0202359. doi: 10.1371/journal.pone.0202359 (PMC6124703; doi:10.1371/journal.pone.0202359)
Supplement: S5 Table — (DOCX) [file pone.0202359.s010.docx]

**S5 Table:** Antihypertensive medication use and risk of all cardiovascular disease and death according to time at target (TITRE) categories.

|  | Antihypertensive medication | |
| --- | --- | --- |
| TITRE categories | Yes | No |
| 0% | 1.0 (reference) | 1.0 (reference) |
| <3 months | 0.43 (0.4,0.46) | 0.88 (0.79,0.98) |
| 3-5.9 months | 0.28 (0.26,0.29) | 0.79 (0.7,0.89) |
| 6-8.9 months | 0.17 (0.16,0.18) | 0.81 (0.71,0.94) |
| 9-11.9 months | 0.1 (0.09,0.12) | 0.56 (0.45,0.7) |

Although higher TITRE was achieved among individuals with antihypertensive medication. The gradient association between TITRE and cardiovascular risk was observed in both groups, suggesting that TITRE is the result of successful blood pressure management strategies, largely contributed by, but not necessarily limited to, antihypertensive medication use only.
