# Supplementary material for: Time spent at blood pressure target and the risk of death and cardiovascular diseases
Source: PLoS One. 2018 Sep 5;13(9):e0202359. doi: 10.1371/journal.pone.0202359 (PMC6124703; doi:10.1371/journal.pone.0202359)
Supplement: S6 Table — (DOCX) [file pone.0202359.s011.docx]

**S6 Table:** Categorical time at target (TITRE) and risk of all cardiovascular disease and death by groups defined by average number of follow-up blood pressure measure categories.^1.2^

|  |  | Average number of follow-up blood pressure measures categories | | | |  |  |
| --- | --- | --- | --- | --- | --- | --- | --- |
|  | All | Q1 | Q2 | Q3 | Q4 | 3 times | 4 times |
| Average annual time at target categories | 169082 | 36862 | 37755 | 37047 | 38578 |  |  |
| All CVD/death |  |  |  |  |  |  |  |
| 0% | 1.0 (Reference) | 1.0 (Reference) | 1.0 (Reference) | 1.0 (Reference) | 1.0 (Reference) | 1.0 (Reference) | 1.0 (Reference) |
| <3 months | 0.58(0.55,0.61) | 0.8 (0.72,0.89) | 0.45 (0.41,0.5) | 0.52 (0.46,0.59) | 0.43 (0.38,0.49) | 0.39 (0.31,0.48) | 0.5 (0.4,0.63) |
| 3-5.9 months | 0.4(0.38,0.42) | 0.59 (0.52,0.68) | 0.36 (0.32,0.4) | 0.32 (0.28,0.36) | 0.29 (0.25,0.33) | 0.3 (0.24,0.37) | 0.35 (0.28,0.44) |
| 6-8.9 months | 0.27(0.25,0.28) | 0.69 (0.57,0.84) | 0.24 (0.21,0.28) | 0.25 (0.22,0.28) | 0.17 (0.15,0.19) | 0.22 (0.18,0.28) | 0.16 (0.13,0.21) |
| 9-11.9 months | 0.17(0.15,0.19) | 0.67 (0.48,0.95) | 0.18 (0.13,0.23) | 0.13 (0.11,0.16) | 0.13 (0.11,0.15) | 0.11 (0.08,0.15) | 0.22 (0.16,0.31) |

^1^Q1: average number of BP measures per year < 0.7 Q2: average number of BP measures per year 0.7-1.6 Q3: average number of BP measures per year 1.6-2.8 Q4: > average number of BP measures per year > 2.8, 3 times: > average number of BP measures per year 2.9-3.4; 4 times > average number of BP measures per year: 3.5-4.4; values unless specified otherwise. ^2^Adjusted for age, gender, year of study entry, multiple deprivation, ethnicity, BMI, smoking, history of diabetes, renal dysfunction, stage two hypertension, total cholesterol, statin use, aspirin use, initial blood pressure lowing drug type, dietary advice, smoking cessation, snapshot ‘control’ status
