# Supplementary material for: Time spent at blood pressure target and the risk of death and cardiovascular diseases
Source: PLoS One. 2018 Sep 5;13(9):e0202359. doi: 10.1371/journal.pone.0202359 (PMC6124703; doi:10.1371/journal.pone.0202359)
Supplement: S7 Table — (DOCX) [file pone.0202359.s012.docx]

**S7 Table:** The extent of missing (%) of case-mix and treatment variables for multivariate models.

|  | Missing, % |
| --- | --- |
|  |  |
| Year of study entry | 0 |
| Age, years | 0 |
| Women | 0 |
| Social deprivation index | 0.34 |
| Smoking (ex, current) | 15.8 |
| Body Mass Index | 53.5 |
| Total cholesterol, mmol/L | 67 |
| History of diabetes | 0 |
| Renal dysfunction | 0 |
| Stage2 hypertension | 0 |
| Intervention |  |
| Initial blood pressure lowering drug class | 0 |
| Baseline aspirin use | 0 |
| Baseline statin use | 0 |
| Dietary advice | 0 |
| Achieving smoking cessation | 0 |
|  |  |
|  |  |

*renal dysfunction: history of renal failure or estimated glomerular filtration rate < 60 mL/min/1.73 m^2^; stage two hypertension: systolic pressure ≥160 mmHg or a diastolic pressure ≥100 mmHg
