# Supplementary material for: Time spent at blood pressure target and the risk of death and cardiovascular diseases
Source: PLoS One. 2018 Sep 5;13(9):e0202359. doi: 10.1371/journal.pone.0202359 (PMC6124703; doi:10.1371/journal.pone.0202359)
Supplement: S8 Table — (DOCX) [file pone.0202359.s013.docx]

**S8 Table:** Categorical time at target (TITRE) and risk of all cardiovascular disease and death in multiple imputed data and complete case analyses.

| Adjusted odds ratio (95% CI) | | | | |
| --- | --- | --- | --- | --- |
|  | | **Original results: imputed dataset** | | **Results from complete case analyses** |
| 0% | | 1.0 (Reference) | | 1.0 (Reference) |
| Missing | | 0.7(0.64,0.77) | | 0.7 (0.64,0.76) |
| <3 months | | 0.58(0.55,0.61) | | 0.57 (0.55,0.6) |
| 3-5.9 months | | 0.40(0.38,0.42) | | 0.39 (0.37,0.41) |
| 6-8.9 months | | 0.27(0.25,0.28) | | 0.26 (0.25,0.28) |
| 9-11.9 months | | 0.17(0.15,0.19) | | 0.17 (0.15,0.19) |
|  |  | |  |  |

^1^Adjusted for age, gender, year of study entry, multiple deprivation, ethnicity, BMI, smoking, history of diabetes, renal dysfunction, stage two hypertension, total cholesterol, statin use, aspirin use, initial blood pressure lowing drug type, dietary advice, smoking cessation, snapshot ‘control’ status.
